# Supplementary material for: Complex‐centric proteome profiling by SEC‐SWATH‐MS
Source: Mol Syst Biol. 2019 Jan 14;15(1):e8438. doi: 10.15252/msb.20188438 (PMC6346213; doi:10.15252/msb.20188438)
Supplement: Supplementary file 8 — Dataset EV7 [file MSB-15-e8438-s008.zip › feature_plots_string/Q9BPX5.pdf]

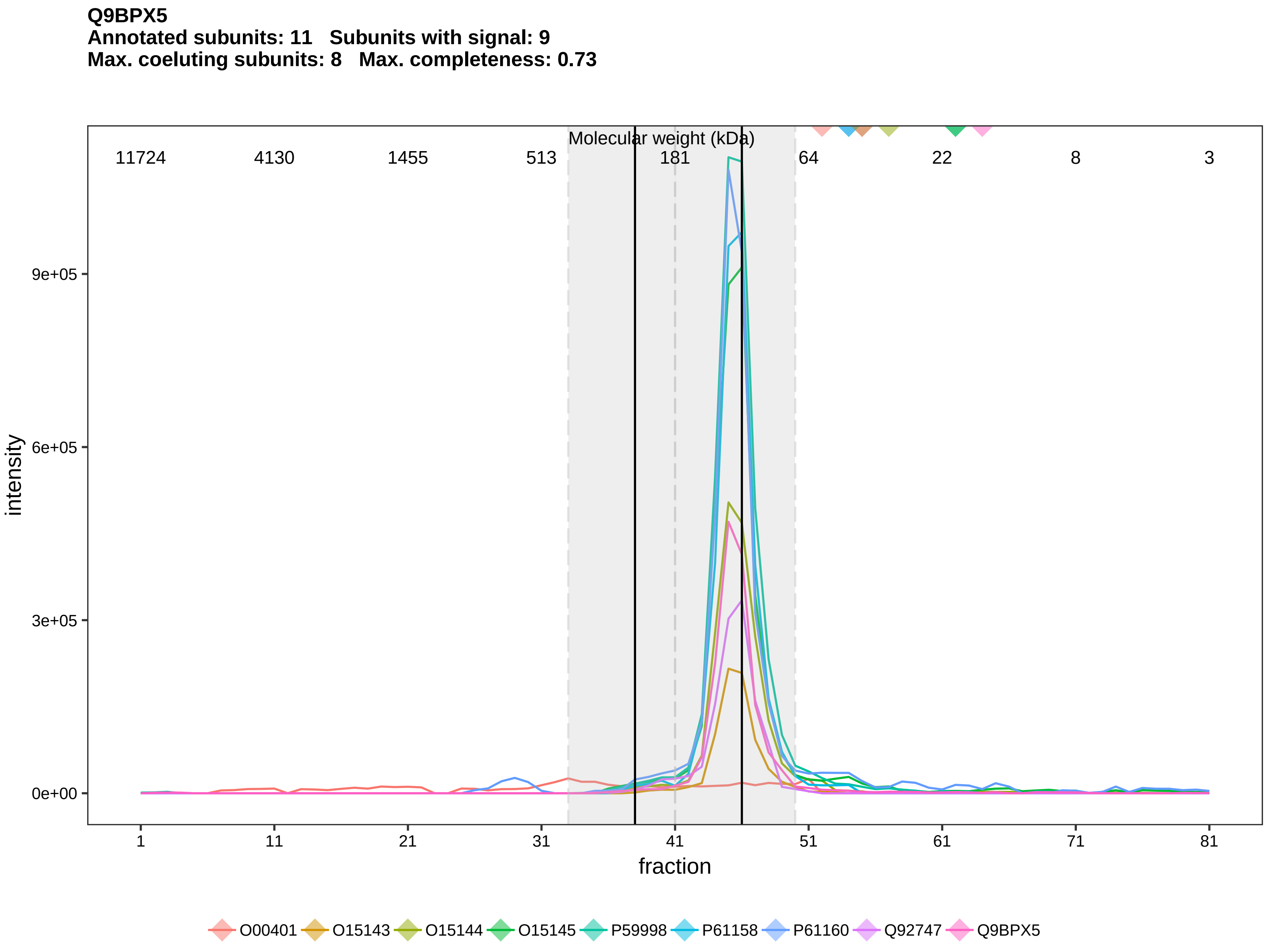
Q9BPX5
Annotated subunits: 11 Subunits with signal: 9
Max. coeluting subunits: 8 Max. completeness: 0.73
Molecular weight (kDa)
11724
4130
1455
513
181
64
22
8
3
9e+05
6e+05
3e+05
0e+00
intensity
1
11
21
31
41
51
61
71
81
fraction
O00401
O15143
O15144
O15145
P59998
P61158
P61160
Q92747
Q9BPX5
